# Case report: a case report of excision of giant lipoma in the posterior neck

**DOI:** 10.3389/fonc.2024.1395130

**Published:** 2024-05-10

**Authors:** Aichao Du, Hongyu Wang, Junqiang Dai, Qiang Dong, Guoqiang Yuan, Yawen Pan

**Affiliations:** ^1^ The Second Clinical Medical College of Lanzhou University, Lanzhou, China; ^2^ Gansu Provincial Key Laboratory of Neurology, Lanzhou, China; ^3^ Department of Neurosurgery, The Second Hospital of Lanzhou University, Lanzhou, China

**Keywords:** lipoma, neck, giant lipoma, surgery, case report

## Abstract

Lipomas, benign tumors originating from the anomalous proliferation of adipocytes, predominantly emerge in regions rich in adipose tissue. However, their presence in the head and neck areas remains rare, constituting approximately 13% of all diagnosed lipoma cases. This study presents a case involving a substantial subcutaneous lipoma located at the posterior neck, measuring about 20 cm × 19 cm × 10 cm. The patient presented with swelling and pain in the back of the neck. And the considerable dimensions of this lipoma significantly impacted the patient’s quality of life and aesthetic appearance. Concurrently, the patient exhibited symptoms indicative of degenerative cervical spine disease and cervical disc herniation. After admission, a comprehensive examination, including ultrasound, CT scan, and MRI, was conducted. Given the clinical complexity, the decision for surgical intervention was deemed essential. The surgical strategy entailed a meticulous total excision of the tumor through an incision made in the posterior cross-neck, coupled with the strategic removal of excess skin. To facilitate wound healing, postoperative management included the use of negative pressure drainage. Pathological examination conclusively identified the mass as a lipoma. Postoperative follow-ups indicated successful recovery, as evidenced by the restoration of the neck’s aesthetic contour and the complete resolution of the previously observed restrictions in sagittal neck movement.

## Introduction

Lipomas, benign tumors frequently arising from the abnormal proliferation or accumulation of adipocytes, are commonly found in regions with a high concentration of fat. These tumors are more prevalent in individuals aged 40-50 years, with a higher incidence in males than in females. Approximately 13% of lipomas occur in the head and neck area, also affecting the upper and lower limbs and the trunk (1, 2). Characterized by slow growth, lipomas typically present as movable, painless, and non-indurated masses. A lipoma is classified as giant when its dimensions exceed 10 cm in diameter or its weight exceeds 1000 g (3).

## Case description

**Figure 1:**
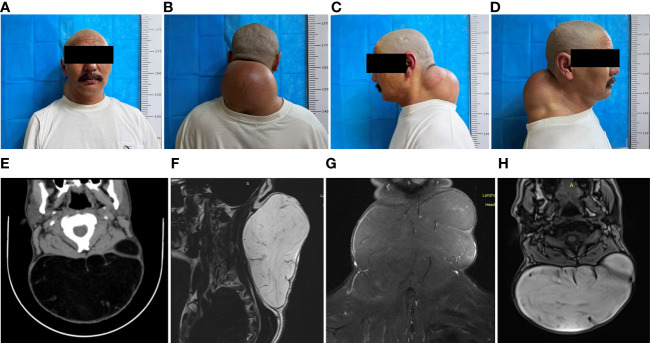
Preoperative clinical and imaging presentation of a giant liposarcoma of the neck. **(A)** Preoperative anterior section. **(B)** Preoperative dorsal view. **(C, D)** Preoperative lateral views. **(E)** Preoperative CT of the neck shows a large posterior cervical mass with clear borders, measuring approximately 162 × 92 × 166 mm, with a CT value of approximately -108 HU. Separation shadows are seen within the mass. **(F–H)** MRI of the cervicothoracic segment showed a large cervical mass with a double-high signal, reduced T2-fs signal, and a linear separation signal shadow in the neck; a linear enhancement shadow was seen on DWI. Cervicothoracic MRI showed a large cervical mass with double high signals in the cervical spine, reduced T2-fs signals, and linearly separated signal shadows, measuring approximately 166×17.6×10.7 cm, with low signals on DWI and linear enhancement on the enhancement scan. The cervical spine was normal with physiological curvature; a mass-like T2-FS high signal shadow was seen in the spinal cord at the C2 level.

A non-tender, movable mass in the neck, initially measured at approximately 5 cm x 5 cm x 3 cm a decade ago, was present in a 47-year-old male patient without any treatment. Over the last ten years, the mass has experienced a gradual increase in size, currently measuring about 20 cm × 19 cm × 10 cm, as depicted in (**Figures 1A–D**). This significant growth severely limited the range of sagittal motion of the cervical spine, adversely affecting the patient’s aesthetic appearance and lifestyle choices. For the past 10 days, the patient has reported experiencing swelling and discomfort in the posterior neck area.

The posterior neck appeared elevated with a deep red skin tone upon examination at the time of admission, though the skin temperature remained within normal limits. Palpation revealed a mass of approximately 20 cm × 19 cm × 10 cm with a smooth surface, clearly demarcated from the surrounding tissues, moderately soft in texture, and displaying typical mobility. No pulsation was detected, and there were no signs of compression pain or attachment to the overlying skin. Imaging was subsequently performed. Through ultrasound examination, a hyperechoic mass measuring approximately 16.6×17 cm was identified beneath the skin of the posterior neck. This mass exhibited a uniform shape, clear boundaries, irregular internal echogenicity, and no signs of blood flow, consistent with the characteristics of a lipoma. Computed tomography (CT) of the cervical area revealed a distinct and sizable mass, with dimensions approximating 162×92×166 mm, with a CT density nearing -108 Hounsfield Units (HU). The visualization of specific internal shadows further supported the diagnosis of a giant neck lipoma (**Figure 1E**). Magnetic Resonance Imaging (MRI) of the cervicothoracic segment highlighted a significant presence in the cervical region, characterized by two high-signal intensity shadows, a diminished T2-fs signal, and a linearly distinct signal shadow. The lesion, measuring about 16.6×17.6×10.7 cm, displayed a low signal on Diffusion-Weighted Imaging (DWI) and a pronounced linear shadow on enhancement scanning. The cervical spine maintained its normal features and physiological curvature. Additionally, a distinct T2-fs high-signal nodular shadow was observed in the spinal cord at the C2 level, corresponding to the typical features of a lipoma (**Figures 1F–H**).

**Figure 2:**
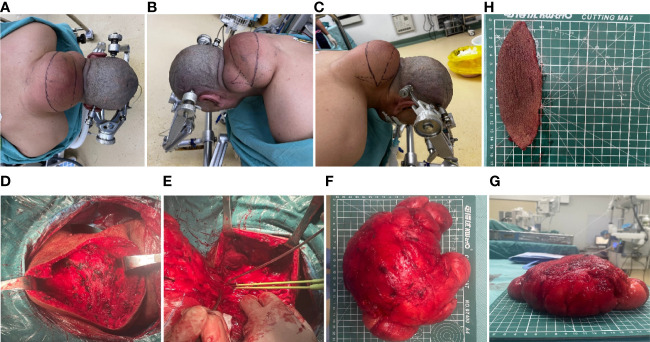
Intraoperative clinical images. **(A–C)** Preoperative design of transverse incision and trimming of the shuttle flap. **(D–G)** Intraoperative clinical views of the intact mass. **(H)** Rejected shuttle flap.

**Figure 3:**
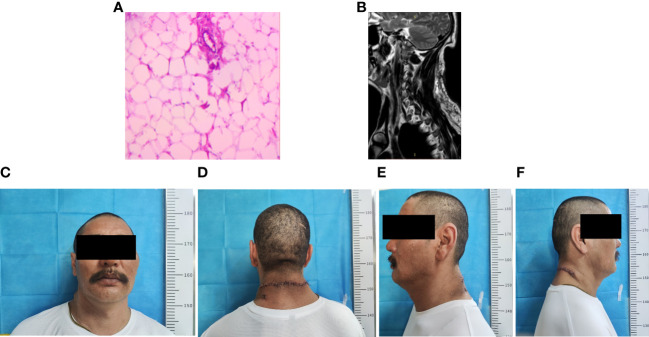
Postoperative clinical and imaging findings. **(A)** Postoperative pathological findings **(B)** Postoperative neck MRI showing complete excision of the subcutaneous neck mass. **(C–F)** Postoperative clinical images of the patient.

The outcomes of the imaging studies initially indicated the presence of a lipoma, leading to the arrangement of a lumpectomy under general anesthesia. A transverse incision was made on the posterior aspect of the neck (**Figures 2A–C**), followed by a skin incision that exposed a large mass, encapsulated mainly by an intact outer membrane. Using tissue scissors, the periphery of the tumor was carefully delineated, revealing lobulated structures in the deeper and outer layers, which were densely packed with fibrous connective tissue of a firm consistency. These scissors facilitated the detachment of the tumor from its adjacent tissues, ensuring a comprehensive removal of the mass. Subsequent to the removal of the pathological tissue for analysis (**Figures 2D–G**), hemostasis was achieved, excess skin was excised according to a predetermined plan (**Figure 2H**), and the site was closed with sutures, followed by the insertion of a negative pressure drainage tube. Macroscopically, the excised specimen presented a greyish-brown appearance, measuring 20×18×8 cm. The specimen’s envelope remained intact, and the cut surface displayed a grayish-yellow color. Microscopic examination revealed a predominance of mature adipose tissue within the tumor. Notably, the adipocytes exhibited homogeneity without significant variation, and a thin fibrous capsule was observed encasing the tumor. Consequently, the final diagnosis of a large subcutaneous lipoma in the neck region was established (**Figure 3A**). Postoperative recovery proceeded without complications, and a follow-up MRI confirmed the complete excision of the mass (**Figure 3B**). The aesthetic outcome of the neck was exceptionally pleasing (**Figures 3C–F**), with a full restoration of mobility. Following thorough deliberations at a multidisciplinary meeting, the patient was discharged on the sixth day post-operation. At a follow-up visit three months later, the patient had fully recovered, with no recurrence of the mass observed.

## Discussion

The literature on giant lipomas, especially those located in the posterior neck region, is limited, with the mechanisms underlying their development remaining largely elusive. Some theories propose a correlation between the formation of lipomas and prior injury (4). Alternatively, various hypotheses have been put forward, linking the occurrence of lipomas to conditions such as Madelung’s disease, dermatophytosis, and genetic factors. Additionally, there is speculation regarding the influence of Cushing’s disease and certain HIV-associated lipodystrophies on the pronounced fat accumulation observed in the neck area (5). The intricate interplay among these factors in the development of giant neck lipomas underscores the complexity of this condition and calls for further investigation and understanding.

Identifying giant lipomas presents significant challenges due to their substantial size, necessitating meticulous exclusion of malignancies and nuanced differentiation from liposarcomas, malignant tumors arising from primitive mesenchymal cells. Liposarcomas exhibit marked tissue heterogeneity and varied differentiation levels, complicating the diagnostic process (6, 7). Because liposarcoma has high local recurrence rates, accurate preoperative differentiation between lipoma and liposarcoma is critical for planning the appropriate surgical resection margins. Clinical manifestations, imaging, and pathology can help to distinguish between the two, with notable differences observed in patient ages and the tumor’s location, depth, and size, along with other crucial aspects of clinical manifestation. Clinically, in terms of the mean age, lipoma patients are generally younger than those with liposarcoma (8). Lipomas can appear in various body parts, particularly the upper limbs and the abdominal wall/trunk region. Most lipomas are located on the surface of the subcutaneous tissue, with only a minority extending deeper into the subpleural fascia or the intermuscular or intramuscular spaces of limbs. Approximately 85% of superficial lipomas are less than 5 cm in diameter. In contrast, liposarcomas typically manifest in deeper anatomical layers, with tumors often exceeding 11 cm in diameter at initial detection, thus exerting pressure on adjacent structures and internal organs (9, 10). Diagnostic imaging, including ultrasound, Computed Tomography (CT), and Magnetic Resonance Imaging (MRI), plays a pivotal role in differentiating these tumors. Ultrasound, while the primary diagnostic tool, has limited efficacy due to the similar hypoechoic and heterogeneous internal echogenic characteristics shared by both tumors (11). CT with venography is crucial in identifying liposarcoma. Although both lipoma and liposarcoma typically exhibit low CT attenuation values, ranging from -10 HU to -150 HU, this imaging modality is exceptionally valuable for delineating the spatial relationships between the tumor and adjacent displaced visceral organs (10). MRI excels with its high sensitivity, differentiating lipomas from liposarcomas through features such as encapsulated, uniform fat masses that emit strong signals on both T1- and T2-weighted images, akin to subcutaneous fat. Indications of liposarcoma on MRI include thickened or nodular septa (over 2 mm), lesions with intense T2 signals, and significant enhancement areas (12). In histopathological evaluations, a fine-needle aspiration biopsy is often required to differentiate between lipomas and liposarcomas, particularly for lesions deeper than the subfascial plane and larger than 5 cm, necessitating further tissue biopsy (13). For tumors smaller than 5 cm, an excisional biopsy is often appropriate. However, in ambiguous cases, less invasive biopsy techniques are preferred to reduce the risk of complications from positive surgical margins. Challenges such as inadequate sample sizes for fine-needle aspiration and improper biopsy site selection can impede the accurate diagnosis of liposarcoma (10). Ultrasound-guided puncture has improved the diagnostic accuracy of biopsies for soft tissue tumors (14). Surgical excision remains the definitive treatment for certain larger tumors, whether they are lipomas or liposarcomas. Immunohistochemical staining and Fluorescence in Situ Hybridization (FISH) are critical tools for differentiating between these two. Liposarcoma is particularly characterized by the amplification of the chromosome 12q13-15 region, which impacts the MDM2 gene. The absence of MDM2 staining effectively excludes the diagnosis of liposarcoma (15). While FISH for MDM2 and CDK4 gene amplification is considered the gold standard for distinguishing between atypical lipomatous tumors/well-differentiated liposarcomas (ALT/WDLS) and lipomas, the FISH assays can take days or even weeks to complete. Additionally, not all medical facilities have the specialized equipment and reagents necessary for FISH analysis. In contrast, immunohistochemical staining is widely available at many institutions and provides a reliable means for the accurate differentiation between benign and malignant tumors (16, 17).

Treatment options for lipomas include surgical and non-surgical methods, with the latter encompassing steroid injections and liposuction aimed at reducing the lipoma’s size and removing fat tissue (18). However, given the size of the lipoma in this case, surgical excision is preferred. Especially important is the complete removal of the lipoma’s capsule to prevent recurrence. The surgical approach adopted here was successful, with no tumor recurrence observed in subsequent follow-ups. Critical to the surgical procedure is the strategic planning of the incision and flap design. Considerations for such procedures encompass: (1) the design of an incision that provides full exposure of the mass, thereby facilitating surgical operation and ensuring complete tumor resection, given the mass’s substantial size; (2) the maintenance of adequate blood flow to the flap during mass separation to avert flap necrosis and failures in postoperative wound healing; (3) the flap’s design aimed at correcting neck deformity and enhancing aesthetic results; (4) the challenge of applying pressure postoperatively, considering the mass’s location at the back of the neck, and the management of the large postoperative cavity through the use of negative pressure drainage to optimize wound healing. In this case, a transverse incision was meticulously executed on the posterior neck, revealing the extensive lipoma. The local skin, affected by prolonged friction with clothing, exhibited sparsity, capillary congestion, hyperplasia, and hyperpigmentation. A spindle flap was thoughtfully designed for the resection, with a focus on aesthetic improvement and the reduction of malignant potential. Postoperatively, the wound demonstrated excellent healing, yielding aesthetically pleasing results. Given the lump’s size, the post-surgical cavity was notably large. While ensuring proper hemostasis to mitigate bleeding risks, it is crucial to be vigilant for potential complications such as hematoma, wound infection, and keloid formation. To facilitate optimal wound healing, a negative pressure drainage system was employed to manage the exudate. The patient was successfully discharged, and follow-up over three months showed significant improvement in the patient’s quality of life, with beautifully contoured neck lines and no local discomfort.

## Conclusion

Giant lipomas located in the head and neck region, although rare, possess the potential to considerably impact both functionality and aesthetics, despite their benign nature. In such instances, conducting a comprehensive imaging assessment is critical to evaluating surgical risks accurately. It is imperative to meticulously consider surgical alternatives. Following the intervention, a detailed histopathological analysis of the specimens becomes essential to exclude the possibility of malignancy, thereby guaranteeing patient safety. This case contributes new insights and addresses an existing gap in the literature regarding similar instances.

## Data availability statement

The original contributions presented in the study are included in the article/supplementary material. Further inquiries can be directed to the corresponding author.

## Ethics statement

The studies involving humans were approved by Ethics Committee of the Second Hospital of Lanzhou University. The studies were conducted in accordance with the local legislation and institutional requirements. The participants provided their written informed consent to participate in this study. Written informed consent was obtained from the individual(s) for the publication of any potentially identifiable images or data included in this article.

## Author contributions

AD: Investigation, Visualization, Writing – original draft, Writing – review & editing. HW: Visualization, Writing – review & editing. JD: Writing – review & editing. QD: Writing – review & editing, Visualization. GY: Supervision, Writing – review & editing. YP: Supervision, Writing – review & editing.
